# Protease Enzyme Inhibitor Cream for the Prevention of Diaper Dermatitis After Gastrointestinal Surgery in Children: Lessons Learned from a Randomized Controlled Trial

**DOI:** 10.3390/children12081028

**Published:** 2025-08-05

**Authors:** Demi Huijgen, Irene K. Schokker-van Linschoten, Hendt P. Versteegh, Johanneke G. H. Ruseler-van Embden, Leo M. C. van Lieshout, Jon D. Laman, Cornelius E. J. Sloots

**Affiliations:** 1 Department of Pediatric Surgery, Erasmus University Medical Center, Sophia Children’s Hospital, 3015 GD Rotterdam, The Netherlands; i.vanlinschoten@erasmusmc.nl (I.K.S.-v.L.); h.versteegh@erasmusmc.nl (H.P.V.); c.sloots@erasmusmc.nl (C.E.J.S.); 2 Department of Immunology, Erasmus University Medical Center, 3015 GD Rotterdam, The Netherlandsj.d.laman@umcg.nl (J.D.L.); 3 Department of Pathology and Medical Biology, University Medical Center Groningen (UMCG), University Groningen, 9713 GZ Groningen, The Netherlands

**Keywords:** diaper rash, dermatitis, prevention, gastrointestinal surgery, protease inhibitors

## Abstract

**Highlights:**

**What are the main findings?**

- Diaper dermatitis occurs in 73.1% of children after gastrointestinal surgery.
- Diaper dermatitis develops within the first two weeks following surgery.

**What are the implications of the main findings?**

- Diaper dermatitis is a significant clinical issue that requires increased awareness and attention in both research and practice.
- Although no superior preventive strategy was identified, the study offers valuable epidemiological and methodological insights to enhance future investigations.

**Abstract:**

**Background**: Diaper dermatitis (DD) frequently occurs following pediatric gastrointestinal surgery and may lead to severe morbidity despite preventive measures. This study aims to evaluate the effectiveness of potato-derived protease enzyme inhibitor cream (PPEIC) in preventing DD after gastrointestinal surgery in children. **Methods**: In this double-blinded, single-center RCT, 30 patients under three years of age undergoing gastrointestinal surgery were randomized 1:1 to prevention using PPEIC or Panthenol cream (PC). The creams were applied after each diaper change for four weeks postoperatively. At two and four weeks, two observers evaluated photographs of the perianal region for the presence and severity of DD. The primary outcome was the severity of DD four weeks after surgery. **Results**: From November 2020 to March 2023, 30 patients were included. Two patients withdrew directly after randomization, resulting in 13 PPEIC and 15 PC patients. In total, nineteen patients (73.1%) developed DD—eight (66.7%) in the PPEIC group and 11 (78.6%) in the PC group (*p* = 0.665)—of whom twelve (63.2%) suffered severe DD. All DD cases developed within the first two weeks, resulting in half of the patients discontinuing the preventive cream before the four-week endpoint. **Conclusions**: This study highlights the significant issue of DD after gastrointestinal surgery, which affects 73.1% of diapered children despite prevention with PPEIC or PC. Although the study was unable to identify a superior preventive method, it offers valuable insights and goals for future research.

## 1. Introduction

Diaper dermatitis (DD) is an inflammatory condition that affects the perianal, gluteal, and genital skin, caused by exposure to feces and urine [1]. It burdens children with excessive crying, agitation, and disrupted eating and sleeping patterns due to pain [2]. The incidence of DD in healthy neonates and infants ranges from 25 to 65 percent [3] and is expected to be higher after gastrointestinal surgery. Prevention of DD includes frequent diaper changes and topical treatment with creams such as panthenol, zinc oxide paste, petroleum jelly, or barrier cream [2]. While some studies have evaluated these measures, the limited number of clinical trials and wide range of reported efficacy results in a lack of clarity regarding their overall efficacy [4,5].

DD is primarily caused by skin irritation from exposure to proteases secreted by the pancreas, small intestinal brush border, and stomach [2,4,6,7,8,9]. Normally, these proteases are neutralized as they transit along the small bowel and colon. However, in conditions with reduced intestinal transit time, such as after colectomy, there is a decrease in the inactivation of intestinal proteases, resulting in feces with high proteolytic activity. Upon contact with the perianal skin, these enzymes can degrade the epidermis and underlying tissue, contributing to the development of DD [8]. Given this etiology, inhibiting protease activity is hypothesized to help prevent and treat DD.

Among the various sources of protease inhibitors, potato tubers are a particularly promising source as they contain a high concentration of proteases, which account for about 50% of the total soluble proteins found in potato juice. Additionally, unlike most purified inhibitors from other sources, which usually target a single enzyme, the potato inhibitor fraction displays a broad range of activity, capable of inactivating nearly all human intestinal proteases [10]. The potential of these potato-derived protease inhibitors has been illustrated in a pilot study on four patients with severe DD after colon resection for Hirschsprung’s Disease, which showed that treatment with a potato-derived protease inhibitor cream (PPEIC) significantly improved the perianal skin condition [6]. However, the efficacy of PPEIC in preventing DD after gastrointestinal surgery has not yet been studied. This randomized controlled trial aims to investigate the effectiveness of PPEIC in preventing DD after gastrointestinal surgery in children.

## 2. Materials and Methods

### 2.1. Study Design

A single-center, double-blind, randomized controlled trial was conducted between November 2020 and March 2023. Diaper-wearing patients under three years of age undergoing gastrointestinal surgery (e.g., ileostomy reversal, colostomy reversal, or pull-through surgery in patients unaccustomed to diapered feces due to reliance on rectal irrigation) were eligible for inclusion. Exclusion criteria were pre-existing DD and allergies. Additionally, patients with parents who were unable to provide informed consent due to language barriers were not enrolled in the study. The study adheres to the tenets of the Declaration of Helsinki, and the institutional Ethical Committee approved the study protocol (MEC-2020-0204). Informed consent was obtained from the patient’s parents.

### 2.2. Study Procedures and Data Collection

Eligible patients were preoperatively approached by their pediatric surgeon. After surgery, participating patients were randomly assigned to either the intervention group or the control group using an online random number generator. Patients in the intervention group received preventive care using a PPEIC (Diapocare^®^, manufactured at EuroDrug Laboratories, The Hague, The Netherlands), containing 5% naturally derived protease enzyme inhibitor extracted from non-genetically modified potatoes. Patients in the control group received the study center’s standard preventive care, which included Panthenol cream (PC, Bepanthen^®^, manufacturer’s details are the same as those of Diapocare^®^) containing 5% Panthenol as the active ingredient. During the packaging process at the pharmacy, the correct study medication was matched with the corresponding inclusion number. The medication was then dispensed and delivered in neutral packaging, ensuring the blinding of both the participants and investigators. Efforts were made to closely match the study creams in color, odor, and consistency to maintain the blinding method. Additionally, investigators did not participate in the application of the creams at any point during the study. Before receiving the study medication, parents were provided with instructions on how to correctly apply the cream after each diaper change for four weeks post-surgery.

Parents were asked to send photographs of the perianal region two and four weeks after the surgery. Two investigators individually assessed these photographs for DD, using a scoring system ranging from zero to six, as outlined by Buckley et al. [11]. In cases of discrepancies, a consensus meeting was held to agree upon a final score. A score of zero was interpreted as no dermatitis, one to three as mild to moderate dermatitis, and four to six as severe dermatitis.

In cases where DD symptoms caused a significant burden, parents were allowed to discontinue the study cream after consulting with their surgeon or nurse practitioner, to allow for standard treatment with either a barrier cream or zinc-based ointment. Upon discontinuation, additional photographs were taken. The follow-up for these patients continued according to standard hospital protocol and was no longer included in the study.

Baseline characteristics and data on the development of DD were systematically entered into a database. Once all participants had completed the study and data completeness was verified, the database was locked. The treatment allocation code was kept in a sealed envelope inside a locked safe, and the blinding code was revealed only after the database was secured.

The primary outcomes were the presence and severity of DD four weeks after surgery. Secondary outcomes included adverse events related to the PPEIC or PC and postoperative complications within the first four weeks after surgery, classified according to Clavien-Madadi [12].

### 2.3. Statistical Analysis

Although exact numbers are unknown, from our experience, DD occurs in about 90% of diaper-wearing children after gastrointestinal surgery. We expected a minimum of 50% reduction in the severity of DD after four weeks of prevention with PPEIC. A simulation-based power analysis was conducted with a two-sided significance level of 0.05 for the effect of 50%. To achieve a power of 80%, 30 participants were needed, with 15 participants in each trial arm.

Statistical analysis was performed in SPSS (IBM SPSS Statistics for Windows, version 28, released 2021). The objective was to compare the severity scores four weeks after the start of preventive care using a general linear mixed model approach. Further outcomes, including the incidence of DD and the duration of study cream use, were compared using the Mann-Whitney U test for continuous variables and the Chi-square test or Fisher’s exact test for categorical variables. Data are presented as medians and interquartile ranges unless stated otherwise. The level of statistical significance was set at *p*  <  0.05.

## 3. Results

### 3.1. Population and Trial Course

**Figure 1:**
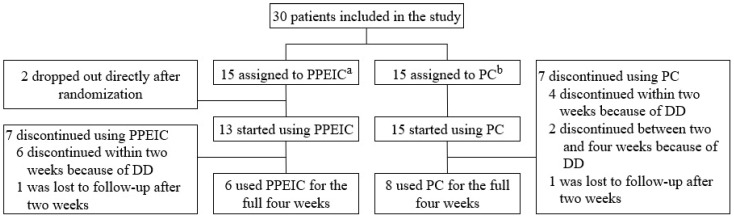
Trial Course. ^a^ PPEIC: potato-derived protease enzyme inhibitor cream, ^b^ PC: panthenol cream.

Figure 1 provides a flowchart of the trial course. From November 2020 to March 2023, 30 patients were assigned to the PPEIC group or the PC group. After two withdrawals directly after randomization, the PPEIC group consisted of 13 patients, and the PC group of 15 patients. Table 1 shows the baseline characteristics, including underlying diseases and types of surgeries performed, as well as postoperative complications observed in both groups. The two groups did not differ in terms of sex, gestational age, birth weight, age at inclusion, or 30-day postoperative complication rate. One patient in the PPEIC group presented with a vaginal (and possible perineal) candidiasis, which was successfully treated with Miconazole. None of the other complications were related to the study creams.

Twelve patients (42.9%), six from each group, discontinued using the study cream before the four-week endpoint after consulting their surgeon due to DD symptoms (Figure 1). There were no significant differences in the days to discontinuation between the PPEIC group (median 8.5 days, IQR 5.75–12.75) and the PC group (median 8.5 days, IQR 6.75–20) (U = 14.5, z = −0.563, *p* = 0.573). Furthermore, two patients, one from each group, were lost to follow-up after two weeks, resulting in missing data on the development of DD for these individuals. In total, six out of 13 patients (46.2%) in the PPEIC group used the study cream for the full four-week period, compared to eight out of 15 patients (53.3%) in the PC group (*p* = 0.705).

### 3.2. Diaper Dermatitis

**Figure 2:**
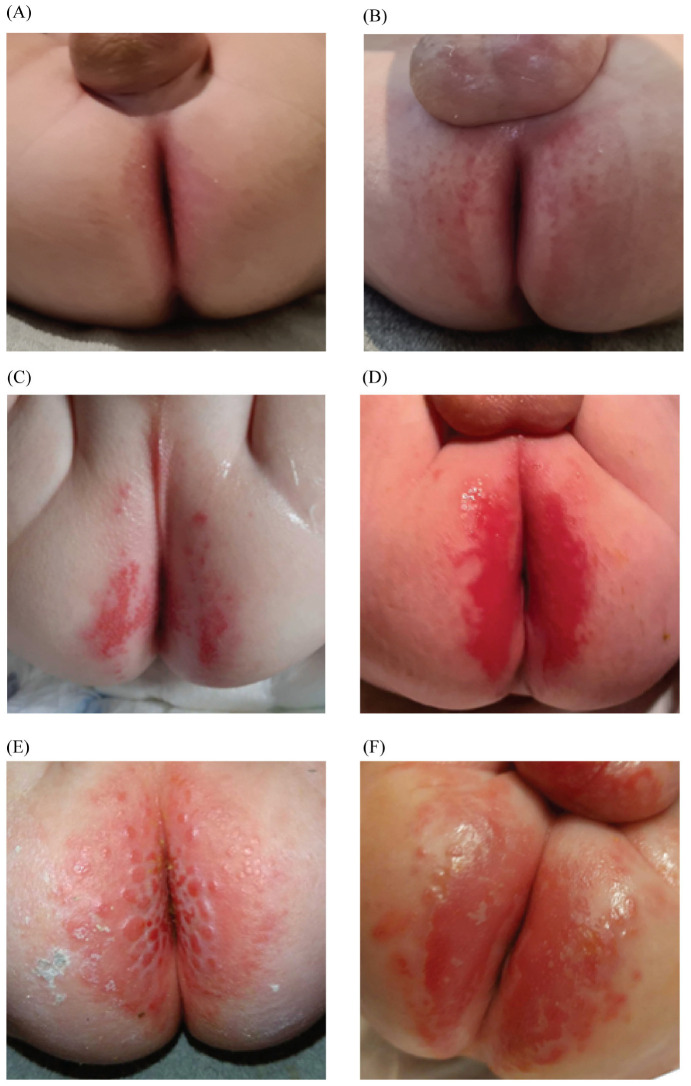
Clinical example for every severity score of DD. Panels (**A**–**F**) show increasing diaper dermatitis severities ranging from 1 to 6.

**Figure 3:**
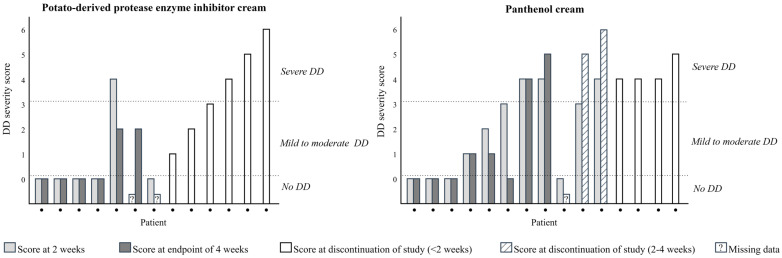
Diaper Dermatitis severity scores for PPEIC and PC stratified by severity and completeness of data. This figure provides an overview of the severity scores of DD at 2 and 4 weeks or at the time of discontinuation for each individual in the PPEIC and PC groups. Each dot represents one patient. The patients are arranged from left to right based on the completeness of their data, with those having complete data on the left and those with incomplete data on the right. They are then further arranged according to the severity of their DD, with those without DD on the left and those with the most severe DD on the right.

Figure 2 provides an example for each severity grade, and Figure 3 provides an overview of the DD severity scores at two and four weeks or at the time of discontinuation. Among the 26 patients with data on DD development, 19 patients (73.1%) developed DD, all of which occurred within the first two weeks of the study. DD was observed across all subgroups of disease entities (Table 2). Of the 19 patients with DD development, 12 (63.2%) suffered from severe DD, and seven (36.8%) from mild to moderate DD. In the PPEIC group, eight out of 12 patients (66.7%) developed DD, compared to 11 out of 14 patients (78.6%) in the PC group (*p* = 0.665). Because of the limited number of patients who used the study cream for the full four-week period, the severity scores measured four weeks after the start of preventive care could not be reliably compared between the groups using the general linear mixed model.

## 4. Discussion

Diaper dermatitis (DD) is a great burden in the postoperative care of children after gastrointestinal surgery, with 73.1% of these children developing DD. Among those affected, 63.2% suffered severe DD, characterized by excessive erythema, skin rash, and blistering. These high incidence and severity rates contributed to a low study completion rate. Nevertheless, the results underscore the need for greater attention to DD and offer valuable insights for future research.

DD is mainly prevented and treated using the ABCDE method: Air (diaper-free time), Barrier (creating a skin barrier in the diaper area), Cleansing (cleaning the diapered area), Diapering (use of superabsorbent diapers), and Education (guiding of caregivers) [13]. Studies of the prevention of DD in the general population focus on several aspects of this ABCDE method [3]. However, no method has demonstrated clear superiority over others, making the prevention and treatment of DD primarily empirical. Moreover, it is unclear to what extent preventive methods used in healthy children are effective in children after gastrointestinal surgery.

This randomized study is the first to focus on preventing DD in children who have undergone gastrointestinal surgery and is one of the few to report on its incidence [14,15,16,17,18]. Most existing studies on postoperative DD primarily focus on children with Hirschsprung’s Disease and do not represent the broader population undergoing gastrointestinal procedures [14,15,16,18]. Additionally, the reported incidence rates vary widely, ranging from 4% to 100%, and severity descriptions are often lacking. Therefore, even though our study could not identify a superior DD prevention method, it does provide valuable data on the incidence and severity of DD in diaper-wearing children following gastrointestinal surgery.

In our study, PPEIC and PC were ineffective in preventing DD. The lack of effect of PPEICs may be explained by the multifactorial etiology of DD. After gastrointestinal surgery, the shortened intestinal transit time increases proteolytic enzyme activity [8,19,20], which is the target of PPEICs. However, additional factors are considered to contribute to DD development in children after gastrointestinal surgery, such as maceration of the stratum corneum from loose stool passage, making the skin more vulnerable to irritants [17]. Moreover, there is a correlation between fecal diversion duration by an enterostomy and DD development, likely due to insufficient water absorption in the unused intestine and prolonged lack of adaptation of the perianal skin to diapered feces. Consequently, only targeting protease activity might not be sufficient to prevent DD in children after gastrointestinal surgery.

Although this study does not provide evidence suggesting that PPEIC is a superior preventive measure, it may be effective in treating (severe) DD. Potato-derived protease inhibitors target a broad spectrum of proteases and have been shown to decrease proteolytic activity in the feces of patients after gastrointestinal surgery [8,10,21,22]. Moreover, in a study of four infants with severe therapy-resistant DD after surgery for Hirschsprung’s Disease, 75% showed improvement in the perianal skin after treatment with PPEIC [6]. Notably, one patient in our study showed a decrease in DD severity score from four to two after continuing PPEIC use, highlighting the need for further research into the role of PPEIC in treating severe DD.

Our research on DD in children who have undergone gastrointestinal surgery presented several challenges that offer valuable insights for future studies. A significant issue was the lack of a uniform definition of (severe) DD and reliable scoring systems for its assessment, as current systems are prone to bias and lack strong content validity [23]. For example, variations in angle, exposure, and photo quality in our study may have influenced scoring results, highlighting the necessity for the development of a robust scoring system and the use of uniformly captured images.

Another challenge arises from the multitude of factors that influence the onset of DD, including diaper type, defecation frequency and consistency, and therapy adherence. Controlling these factors in a study is challenging, as DD primarily arises after hospital discharge. To ensure feasibility and encourage patient participation, we chose a more straightforward study design. However, the severity of the DD issue highlighted by this study emphasizes the need for future large cohort studies that consider the confounding factors mentioned earlier, as well as the duration of fecal diversion via enterostomy and the extent of residual bowel following bowel resection.

Finally, three methodological challenges were encountered. Firstly, the lack of incidence data forced us to rely on estimates when calculating our sample size, which may have resulted in a sample size that was too small for reliable analysis. Secondly, our decision to use an ordinal outcome variable added complexity to the sample size calculation, as methods for estimating effect size and required sample size are more established and straightforward for binary outcomes. However, we believe that simplifying DD severity into a binary variable—only indicating the presence or absence of DD—would have resulted in a significant loss of clinically relevant information. By using a scale to assess the severity of DD, we captured valuable insights regarding both the incidence and severity of the condition, which can facilitate more accurate power analyses in future research. Thirdly, we initially planned to assess both DD presence and severity four weeks after surgery. However, our findings show that DD developed within two weeks in all cases, prompting many parents to discontinue the study cream and start therapeutic treatment. The threshold for discontinuation varied between families; some stopped at relatively low severity scores, while others insisted on continuing despite more severe symptoms. The variability in the duration of preventive cream use and the severity of DD at the point of discontinuation compromised the comparability of severity scores at the four-week mark. To prevent this issue in future research, we recommend assessing DD presence and severity earlier, ideally within the first two weeks post-surgery.

## 5. Conclusions

In conclusion, this study shows that DD poses a substantial problem in children after gastrointestinal surgery, with 73.1% of patients experiencing postoperative DD, of whom 63.2% suffered from severe DD. Although the study was unable to identify a superior preventive measure, it highlights the need for greater attention to DD. Furthermore, it provides valuable insights into the incidence and presentation of DD, which can improve the methodological accuracy of future research. Finally, the study identifies important research objectives, including assessing the effectiveness of PPEIC in treating severe DD following gastrointestinal surgery and developing a comprehensive scoring system for DD.

## Figures and Tables

**Table 1:** Baseline characteristics and postoperative complications of the intention-to-treat population.

	PPEIC ^a^ (n = 13)	PC ^b^ (n = 15)	*p*-Value
Male sex, n (%)	10 (76.9)	10 (66.7)	0.686
Born prematurely (<37 weeks gestational age), n (%)	3 (23.1)	5 (33.3)	0.686
Small for gestational age (<10th percentile), n (%)	1 (7.7)	2 (13.3)	1.000
Age at inclusion in months, median (IQR)	3.2 (2.2–4.5)	4.3 (2.3–6.3)	0.279
Type of surgery and underlying pathology ^c^			
Laparoscopic-assisted pull-through for Hirschsprung’s Disease (no prior ostomy)	3	4	
Ileostomy reversal for Hirschsprung’s Disease	1	2	
Ileostomy reversal and laparoscopic-assisted pull-through for Hirschsprung’s Disease	1	1	
Ileostomy reversal after necrotizing enterocolitis	2	1	
Ileostomy reversal after small bowel atresia	1	2	
Ileostomy reversal after other pathologies ^d^	3	1	
Colostomy reversal after anorectal malformation	2	4	
≥1 post-operative complications, n (%)	6 (46.2)	10 (66.7)	0.274
Type of postoperative complications, n (Clavien Madadi Classification)			
Cholangitis	-	1 (II)	
Prolonged postoperative ileus	-	2 (IB/II)	
Constipation	2 (IB)	2 (IB)	
Fever of unknown origin	2 (IB/II)	4 (II)	
Infection of the ostomy closure wound	-	3 (IB)	
Urinary retention	-	1 (IIIA)	
Edema	-	1 (IB)	
Anastomotic leakage of the ileum	-	1 (IV)	
Persistent diarrhea	1 (IB)	-	
Vaginal candidiasis	1 (IB)	-	
Peripheral intravenous catheter malfunction	1 (IIIA)	-	

Outcomes were compared using the Mann–Whitney U test for continuous outcome variables and the Chi-square test or Fisher’s exact test for categorical outcome variables. ^a^ PPEIC: potato-derived protease enzyme inhibitor cream, ^b^ PC: panthenol cream, ^c^ Groups too small for statistical analysis, ^d^ Other underlying pathologies were colitis, meconium peritonitis, and milk curd syndrome in the PPEIC group and meconium ileus in the PC group.

**Table 2:** Diaper dermatitis development by disease entity.

	Included Patients n = 26 ^a^	DD Development n (%)
Hirschsprung’s Disease	12	9 (75)
Anorectal malformation	5	5 (100)
Necrotizing enterocolitis	3	2 (66.7)
Small bowel atresia	3	1 (33.3)
Other underlying diseases	3	2 (66.7)

^a^ This table includes only patients with data on the development of DD.

## Data Availability

The raw data supporting the conclusions of this article will be made available by the authors on request.

## Abbreviations

The following abbreviations are used in this manuscript:

DD	Diaper dermatitis
PPEIC	Potato-derived protease enzyme inhibitor cream
PC	Panthenol cream
